# Evaluation of the 13-item Hypomania Checklist and a brief 3-item manic features questionnaire in primary care

**DOI:** 10.1192/pb.bp.116.054577

**Published:** 2017-08

**Authors:** Sukhmeet Singh, Paul Scouller, Daniel J. Smith

**Affiliations:** 1 University of Glasgow, Glasgow, UK; 2 NHS Greater Glasgow and Clyde, UK

## Abstract

**Aims and method** The mean delay for bipolar disorder diagnosis is 10 years. Identification of patients with previous hypomania is challenging, sometimes resulting in misdiagnosis. The aims of this study were: (a) to estimate the proportion of primary care patients with depression currently taking antidepressants who have undiagnosed bipolar disorder and (b) to compare a brief 3-item manic features questionnaire with the Hypomania Checklist (HCL-13). The sample comprised patients with a recorded diagnosis of depression, either on long-term antidepressant therapy or with previous multiple courses of antidepressants.

**Results** Of 149 participants assessed, 24 (16.1%) satisfied criteria for bipolar disorder. Areas under the curve (AUC) for the 3-item questionnaire and the HCL-13 were similar (0.79 and 0.72, respectively) but positive predictive values (PPV) were low.

**Clinical implications** Bipolar disorder may be underdiagnosed in primary care. A 3-item questionnaire could be used by general practitioners to screen for bipolar disorder in their patients with depression.

Bipolar disorder affects at least 1–2% of the population and is associated with considerable psychosocial impairment.^1–4^ Accurate diagnosis can be difficult because patients tend initially to present in primary care for help with depression rather than with manic features, and many primary care practitioners do not systematically assess for a history of bipolar disorder.^5–7^ Recently the National Institute for Health and Care Excellence (NICE) recommended that all patients in the UK presenting to primary care with depression should be assessed for a history of manic features, specifically ‘previous periods of overactivity or disinhibited behaviour’.^8^

Recent screening studies of bipolar disorder in UK primary care have identified that between 7 and 10% of individuals with depression may have undiagnosed bipolar disorder, usually bipolar disorder type II.^9,10^ It is also the case that individuals with difficult to treat depression and those with poor response to antidepressants are more likely to have unrecognised bipolar disorder.^11^

In this context, we aimed to estimate unrecognised bipolar disorder within a sample of primary care patients with depression who were taking antidepressant medications, as well as to evaluate the potential clinical utility of two short screening questionnaires for bipolar disorder: the 13-item Hypomania Checklist (HCL-13)^12^ and a brief 3-item manic features questionnaire. Our focus on these brief assessment instruments was stimulated by the need for questionnaires that could be used easily in primary care consultations, which are usually no longer than 10–15 min.

## Method

### Sample

Practice managers of 9 primary care practices in the west of Scotland identified from their databases 2633 patients who (a) had a recorded diagnosis of depression and (b) were either currently taking long-term antidepressants (more than 12 months) or had had 3 or more courses of antidepressants in the past 5 years. Then, 1860 potentially eligible patients were reviewed by their general practitioner (GP) for eligibility and 1833 written invitations were sent by post, with participant information sheets, on behalf of the research team. There were 204 people who responded to this invitation and 151 were interviewed, giving a response rate of 8.23%. The study was approved by the West of Scotland Research Ethics Committee (reference: 13/WS/0071 18th, approval letter dated April 2013).

### Assessment measures

A research nurse completed the Structured Clinical Interview for DMS-IV (SCID-1)^13^ in person in order to obtain a gold-standard diagnosis. An assessment of practice notes was also conducted to corroborate findings from the SCID-1 assessment and to clarify medication regimens. Participants completed the HCL-13 and a brief 3-item bipolar screening questionnaire. This 3-item questionnaire (with a maximum score of 6) was based on the three core diagnostic features for bipolar disorder taken from DSM-5: elevated mood, severe irritability and overactivity.^14^ The questions and scoring were as follows:

1. Have you ever had a period of time when you were feeling so good, ‘high’, excited or ‘hyper’ that other people thought you were not your normal self or you were so ‘hyper’ that you got into trouble? (Definitely no (score 0), perhaps yes (score 1), definitely yes (score 2).)
2. What about a period of time when you were so irritable that you found yourself shouting at people or starting fights or arguments? (Definitely no (score 0), perhaps yes (score 1), definitely yes (score 2).)
3. What about a period of time when you were physically much more active than usual, for example, when you had lots of different projects on the go at the same time? (Definitely no (score 0), perhaps yes (score 1), definitely yes (score 2).)

### Analyses

Analyses included the Student's *t*-test, chi-squared test and chi-squared test for association, and were conducted using SPSS version 21 for Windows. To assess the likely clinical usefulness of the HCL-13 and 3-item questionnaires, in terms of differentiating between bipolar disorder and major depressive disorder (MDD), we calculated sensitivity, specificity, positive predictive value (PPV) and negative predictive value (NPV) using MedCalc and verified these manually. Positive and negative clinical utility measures were calculated using an online calculator constructed by the developer of the MedCalc test (www.psycho-oncology.info/cui.html).^15^

## Results

Of those who had a full diagnostic assessment, two participants were excluded because their SCID results suggested a primary diagnosis of alcohol-related mood problems. In total, 24 participants from our final sample of *n* = 149 had a DMS-IV diagnosis of bipolar disorder (16.1%; 95% CI 10.8–23.2%) and the remainder had a diagnosis of MDD (*n* = 125, 83.9%).

There were no significant differences between the bipolar disorder group and the MDD group in terms of age, gender distribution and socioeconomic status (assessed using the Scottish Index of Multiple Deprivation, SIMD) (Table 1). However, as expected, the bipolar disorder group had higher mean scores on the HCL-13 (9.21 *v*. 6.61, *P*= 0.001) and on the 3-item questionnaire (4.79 *v*. 2.88, *P<* 0.001).

**Table 1:** Characteristics of participants with major depressive disorder (MDD) and bipolar disorder

	MDD (*n* = 125)	Bipolar disorder (*n* = 24)	*P*
Age, years: mean (s.d.)	47.50 (10.50)	47.58 (8.79)	0.972^a^

Females: *n* (%)	77 (62.6)	15 (62.5)	0.992^b^

SIMD 1: *n* (%)^d^ (most deprived quintile)	56 (45.5)	14 (58.3)	0.705^c^

SIMD 2: *n* (%)	20 (16.3)	2 (8.3)	

SIMD 3: *n* (%)	15(12.2)	4(16.7)	

SIMD 4: *n* (%)	13 (10.6)	1 (4.2)	

SIMD 5: *n* (%) (most affluent quintile)	18 (14.6)	3 (12.5)	

HCL-13: mean (s.d.)	6.61 (3.36)	9.21 (2.77)	0.001^a^

3-item questionnaire: mean (s.d.)	2.88 (1.84)	4.79 (1.56)	<0.001^a^

HCL-13, Hypomania Checklist 13; SIMD, Scottish Index of Multiple Deprivation.

a. Student *t*-test.

b. Chi-squared test.

c. Chi-squared test for association.

d. No SIMD data for 1 participant with MDD.

### ROC analyses

**Fig. 1:**
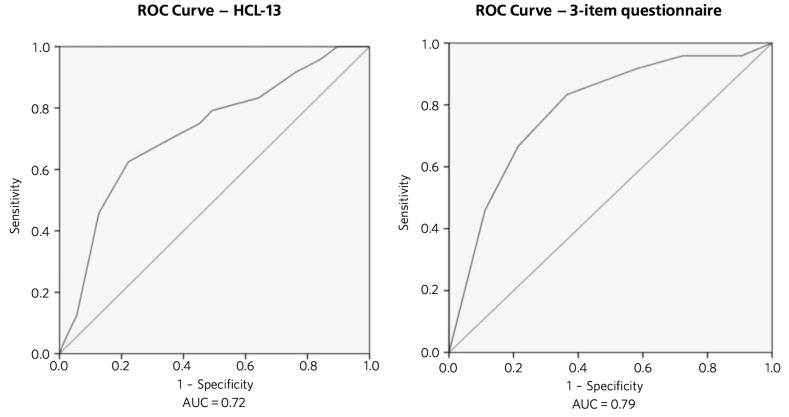
Receiver operating characteristics (ROC) for 13-item Hypomania Checklist (HCL-13) and 3-item questionnaire: discrimination between bipolar disorder (*n* = 24) and major depressive disorder (*n* = 125). AUC (area under curve): 0.72 (95% CI 0.61–0.84) for HCL-13 and 0.79 (95% CI 0.69–0.89) for the 3-item questionnaire.

The receiver operating characteristics (ROC) curves in Fig. 1 demonstrate that both the HCL-13 and the 3-item questionnaire performed well in terms of differentiating between MDD (*n* = 125) and bipolar disorder (*n* = 24). For the HCL-13, an area under the curve (AUC) of 0.72 (95% CI 0.61–0.84) demonstrates a ‘fair’ overall ability of the questionnaire to discriminate effectively between the two groups. Similarly, an AUC of 0.79 (95% CI 0.69–0.89) for the 3-item questionnaire also demonstrates a ‘fair’ overall ability. An AUC of over 0.80 is considered to demonstrate a ‘good’ overall ability to discriminate.^16^

Table 2 presents the sensitivity, specificity, PPV, NPV and likelihood ratio analyses. For the HCL-13, a threshold score of 8 points had a sensitivity of 75.0% and specificity of 55.28% but PPV was low at 24.66%. At a higher threshold of 9 points, the sensitivity was 70.83%, specificity was 63.41%, PPV was 27.42% and NPV was 91.76%. At the lower threshold of 7 points, the sensitivity was 79.17%, specificity was 52.03%, PPV was 24.36% and NPV was 92.75%. Therefore, a threshold of 4 points was chosen to give the best balance between different statistical parameters.

**Table 2:** HCL-13 and 3-item questionnaire screening for bipolar disorder

Questionnaire	Threshold	Sensitivity (%) (95% CI)	Specificity (%) (95% CI)	PPV (95% CI)	NPV (95% CI)	Positive clinical utility (95% CI)	Negative clinical utility (95% CI)
HCL-13	13 points	4.17 (0.11–21.12)	99.19 (95.55–99.98)	50.00 (1.26–98.74)	84.14 (77.16–89.67)	0.021 (0.000–0.317)	0.835 (0.795–0.874)

	⩾12 points	12.50 (2.66–32.36)	95.12 (89.68–98.19)	33.33 (7.49–70.07)	84.78 (77.68–90.33)	0.042 (0.000–0.290)	0.806 (0.762–0.851)

	⩾11 points	45.83 (25.55–67.18)	87.80 (80.68–93.01)	42.31 (23.35–63.08)	89.26 (82.33–94.15)	0.194 (0.000–0.432)	0.784 (0.735–0.833)

	⩾10 points	60.50 (40.59–81.20)	78.05 (69.69–85.01)	35.71 (21.55–51.97)	91.43 (84.35–96.01)	0.223 (0.014–0.432(	0.714 (0.655–0.772)

	⩾9 points	70.83 (48.91–87.38)	63.41 (54.25–71.91)	27.42 (16.85–40.23)	91.76 (83.77–96.62)	0.194 (0.011–0.377)	0.582 (0.509–0.655)

	⩾8 points	75.00 (53.39–90.23)	55.28 (46.06–64.25)	24.66 (15.32–36.14)	91.89 (83.18–96.97)	0.185 (0.013–0.357)	0.508 (0.427–0.589)

	⩾7 points	79.17 (57.85–92.87)	52.03 (42.84–61.12)	24.36 (15.35–35.40)	92.75 (83.89–97.61)	0.193 (0.025–0.361)	0.483 (0.399–0.566)

	⩾6 points	83.33 (62.62–95.26)	36.59 (28.09–45.75)	20.41 (12.93–29.74)	91.84 (80.40–97.73)	0.170 (0.016–0.324)	0.336 (0.237–0.435)

	⩾5 points	91.67 (73.00–98.97)	24.39 (17.10–32.95)	19.13 (12.39–27.52)	93.75 (79.19–99.23)	0.175 (0.032–0.318)	0.229 (0.116–0.341)

	⩾4 points	95.83 (78.88–99.89)	16.26 (10.22–23.99)	18.25 (11.94–26.12)	95.75 (79.19–99.23)	0.175 (0.038–0.312)	0.155 (0.031–0.278)

	⩾3 points	100.00 (87.75–100.00)	15.45 (9.56–23.07)	18.75 (12.40–26.60)	100.00 (82.35–100.00)	0.188 (0.052–0.323)	0.154 (0.029–0.279)

	⩾2 points	100.00 (85.75–100.00)	15.45 (9.56–23.07)	18.75 (12.40–26.60)	100.00 (82.35–100.00)	0.188 (0.052–0.323)	0.154 (0.029–0.279)

	⩾1 point	100.00 (85.75–100.00)	11.11 (6.05–18.25)	18.75 (12.40–26.60)	100.00 (75.29–100.00)	0.179 (0.046–0.312)	0.106 (0.000–0.238)

3-item questionnaire	⩾6 points	45.83 (25.55–67.18)	88.62 (81.64–93.64)	44.00 (24.40–65.07)	89.34 (82.47–94.20)	0.202 (0.000–0.443)	0.792 (0.744–0.840)

	⩾5 points	66.67 (44.68–84.37)	78.05 (69.69–85.01)	37.21 (22.98–53.27)	92.31 (85.40–96.62)	0.248 (0.041–0.455)	0.720 (0.663–0.778)

	⩾4 points	83.33 (62.62–95.26)	64.23 (55.09–72.67)	31.25 (20.24–44.06)	95.18 (88.12–98.67)	0.260 (0.081–0.439)	0.611 (0.541–0.682)

	⩾3 points	91.67 (73.00–98.97)	43.09 (34.20–52.32)	23.91 (15.63 – 33.94)	96.36 (87.47–99.56)	0.219 (0.064–0.375)	0.415 (0.323–0.507)

	⩾2 points	95.83 (78.88–99.89)	28.45 (20.69–37.29)	20.72 (13.61–29.45)	97.22 (85.47–99.93)	0.199 (0.054–0.343)	0.277 (0.169–0.385)

	⩾1 point	95.83 (78.88–99.89)	9.76 (5.14–16.42)	17.16 (11.20–24.63)	92.31 (63.97–99.81)	0.164 (0.031–0.298)	0.090 (0.000–0.223)

HCL-13, 13-item Hypomania Checklist; NPV, negative predictive value; PPV, positive predictive value.

Similarly, a threshold score of 4 on the 3-item questionnaire had a sensitivity of 83.33%, specificity of 64.23% and PPV of only 31.25%. At a higher threshold of 5 points, the sensitivity was 66.67%, specificity was 78.05%, PPV was 37.21% and NPV was 92.31%. At a lower threshold of 3 points, sensitivity was 91.67%, specificity was 43.09%, PPV was 23.91% and NPV was 93.36%. Therefore, a threshold of 4 points was chosen to give the best balance between these different parameters.

The positive clinical utility – the ability of the test to confirm cases of bipolar disorder – was poor for both tests. The negative clinical utility a measure of screening and excluding bipolar disorder, was slightly better for the 3-item questionnaire than the HCL-13 at our threshold values: 0.611 (95% CI 0.541–0.682) compared with 0.582 (0.509–0.655). These thresholds were chosen to give the best balance between sensitivity, specificity, PPV, NPV and positive and negative clinical utility

## Discussion

One of the goals of this study was to estimate how common DMS-IV bipolar disorders might be in a sample of primary care patients taking antidepressant medication, specifically those patients who were either taking antidepressant therapy for more than 12 months or who had had multiple courses of antidepressants over the preceding 5 years. We found that 16.1% of our sample had bipolar disorder. This rate is higher than in previous literature from the UK. In samples of primary care patients, Hughes *et al*^10^ found a prevalence of 7.3% whereas Smith *et al* found a prevalence of 9.6%. Both studies assessed patients with depressive disorder who had been prescribed antidepressant medication. It is possible that the addition in our study of participants who had previously been prescribed multiple courses of antidepressants led to a higher prevalence estimate for bipolar disorder, because unrecognised bipolar disorder is more common in patients with more severe and enduring depression.^17^

We also aimed to compare the HCL-13 and a brief 3-item questionnaire in terms of their ability to differentiate between patients with MDD and bipolar disorder. We found that the AUC for HCL-13 was 0.72, while for the 3-item questionnaire it was slightly higher, at 0.79. For both tests the ability to discriminate between MDD and bipolar disorder in terms of sensitivity and specificity was reasonable, but PPVs were low. This is a function of the low prevalence of bipolar disorder in primary care setting, but represents a potential limitation in terms of the usefulness of these instruments to GPs in everyday clinical practice.^18^ In a review of brief screening instruments for depressive disorder in a low-income country, Hanlon *et al*^19^ concluded that the low PPV at acceptable sensitivity levels may preclude their use in clinical settings.

Nevertheless, we would argue that there may be some use in primary care for these brief screening instruments alongside additional assessments, for example whether patients have a strong family history of mood disorder. The 3-item questionnaire in particular may be useful to GPs in terms of fulfilling the NICE requirement to assess all patients with depression for a history of manic features. The high NPV of 95% means that clinicians may find this useful for excluding a diagnosis of bipolar disorder in their patients with depression. The NICE guidance states that the ideal instrument should be brief, easy to administer and to score, and should be able to be interpreted without extensive and specialist training ^8^

### Strengths and limitations

This was a reasonably large study that took a systematic approach to screening patients in primary care settings. We used definitions of bipolar disorder and MDD based on formal diagnostic classifications by using SCID assessment. The study included a range of people from different social backgrounds, with the majority living in some of the most deprived areas of Scotland. However, it may have been helpful to have more baseline demographic information on patients, such as ethnicity, family history of bipolar disorder and age at onset of depression, and the study may be subject to recall bias because it relied on the patient's recall of prior episodes of manic symptoms rather than a corroborative history. There may also be an issue of selection bias, because GPs were able to exclude certain participants if they felt that they were not suitable for this study As a result of this, and the fact that only one method of recruitment was used in this study, there was a relatively small final sample given the number of invitations sent, which may have led to ascertainment bias. Moreover, the SCID interviewer was not masked to HCL-13 and 3-item scores, which may also have been a source of bias.

Another potential limitation is that the 3-item questionnaire had no requirement for a minimum duration of symptoms. It is possible that individuals with brief periods of affective instability, such as those with borderline personality disorder, would be inclined to respond positively to these questions. Similarly, we did not take a history of alcohol or drug use, and while we did exclude alcohol or substance-induced mood disorders, the use of psychoactive substances could have led to false positives with the 3-item questionnaire.

### Clinical implications

A brief 3-item questionnaire may be clinically useful for GPs who wish to screen for manic features in patients with MDD. This could prompt more detailed assessment, such as an appointment with a relative or friend to obtain a collateral history before assessing the need for a referral to secondary care. Further studies are required in larger samples to assess the clinical usefulness of this test in screening, ideally without the issues of recruitment faced in this study It may also be helpful to develop the 3-item questionnaire further, perhaps with the addition of other items such as the duration of symptoms. While the addition of items would lead to a more statistically sound test, it would also take longer to administer such a test, which may make it less clinically useful.

A proportion of primary care patients with MDD, perhaps as many as 1 in 5, may have undiagnosed bipolar disorder. For busy clinicians working in a time-restricted environment, we suggest that a brief 3-item questionnaire may be a useful screening tool for bipolar disorder and a first step towards a more comprehensive assessment.
